# Crystal structure of (*E*)-*N*′-(5-bromo-2-hy­droxy­benzyl­idene)nicotinohydrazide monohydrate

**DOI:** 10.1107/S2056989015009627

**Published:** 2015-06-03

**Authors:** S. Sravya, S. Sruthy, N. Aiswarya, M. Sithambaresan, M. R. Prathapachandra Kurup

**Affiliations:** a Department of Chemistry, Amrita Vishwa Vidyapeetham, Clappana PO, Kollam 690 525, India; b Department of Applied Chemistry, Cochin University of Science and Technology, Kochi 682 022, India; c Department of Chemistry, Faculty of Science, Eastern University, Chenkalady, Sri Lanka

**Keywords:** crystal structure, aroylhydrazone, carbohydrazide, nicotinohydrazide, hydrogen bonding

## Abstract

In the title compound, C_13_H_10_BrN_3_O_2_·H_2_O, the conformation about the azomethine double bond is *E*. The mol­ecule exists in the amido form with a C=O bond length of 1.229 (2) Å. There is an intra­molecular O—H⋯N hydrogen bond forming an *S*(6) ring motif. The whole mol­ecule is almost planar, with an r.m.s. deviation of 0.021 Å for all non-H atoms, and the dihedral angle between the planes of the pyridine and benzene rings is 0.74 (12)°. In the crystal, the water mol­ecule of crystallization links the organic mol­ecules *via* Ow—H⋯O, Ow—H⋯N and N—H⋯Ow hydrogen bonds and short C—H⋯Ow contacts, forming sheets lying parallel to (100). Within the sheets there is a weak π–π inter­action involving the pyridine and benzene rings [centroid-to-centroid distance = 3.8473 (15) Å]. The sheets are linked *via* C—H⋯Br inter­actions, forming a three-dimensional network.

## Abstract 2

The title compound, has an *E* conformation about the C=N bond and the mol­ecules is planar (r.m.s. deviation for all non-H atoms = 0.021 Å). In the crystal, the lattice water mol­ecule (Ow) links the mol­ecules *via* Ow—H⋯O, Ow—H⋯N and N—H⋯Ow hydrogen bonds, forming sheets lying parallel to (100).

## Chemical context   

Aroylhydrazones can coordinate to transition metals either in the amido form (Bessy Raj & Kurup, 2007 ▸) or in the imino­lato form (Ghosh *et al.*, 2005 ▸; Galić *et al.*, 2011 ▸), leading to the formation of two types of complexes. Hydrazones derived from isonicotinoyl hydrazides are potential drugs for the treatment of the iron-overload associated diseases (Macková *et al.*, 2012 ▸). They are associated with a broad spectrum of biological activities, and studies have shown that nicotinic acid hydrazones could be considered as anti-inflammatory and analgesic agents (Navidpour *et al.*, 2014 ▸; Kheradmand *et al.*, 2013 ▸) and as a novel pharmacophore in the design of anti­convulsant drugs (Sinha *et al.*, 2011 ▸). Hydrazones have been used in chemical processes, in non-linear optics and as sensors as well as in catalytic processes (Hosseini-Monfared *et al.*, 2013 ▸; Du & Hong, 2014 ▸). Their potential as analytical reagents (Galić *et al.*, 2011 ▸) and their uses as mol­ecular switches, metallo-assemblies and sensors have also been reported (Su & Aprahamian, 2014 ▸). Salicyl­aldehyde isonicotinoylhydrazone has also been used for the spectrophotometric determination of gallium(III) and indium(III) (Reddy *et al.*, 2011 ▸).
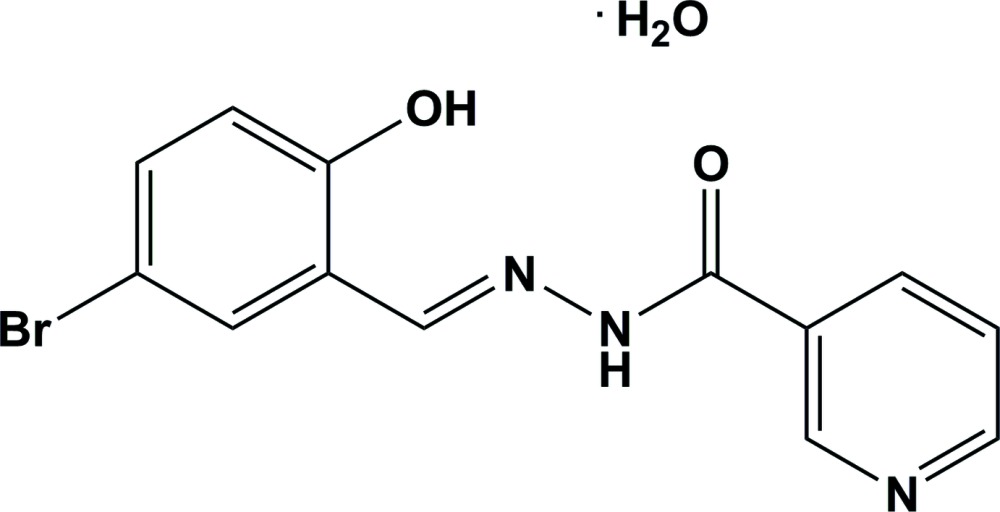


## Structural commentary   

**Figure 1:**
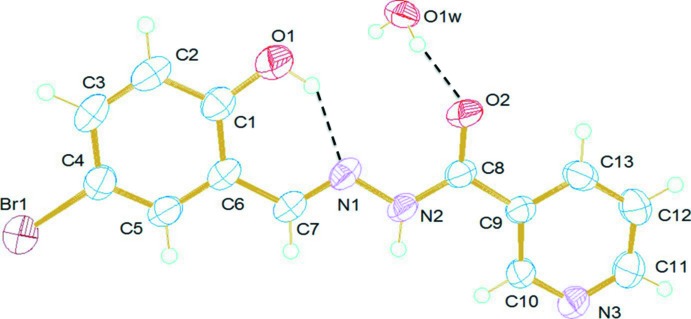
A view of the mol­ecular structure of the title compound, showing the atom labelling. Displacement ellipsoids are drawn at the 50% probability level.

The title compound, Fig. 1 ▸, exists in the amido form with a C8=O2 bond length of 1.229 (2) Å. The mol­ecule has an *E* conformation with respect to the azomethine bond, which is confirmed by the torsion angle C6—C7=N1—N2 of 179.09 (19)°. The two aromatic rings (C1–C6 and N3/C9–C13), are inclined to the almost planar hydrazone moiety [O2/C8/N2/N1/C7; planar to within 0.006 (2) Å] by 2.12 (9) and 1.40 (8)°, respectively, and to each other by 0.74 (12)°. There is an intra­molecular O—H⋯N hydrogen bond present in the mol­ecule that involves the phenolic oxygen, O1 and the azomethine nitro­gen atom, N1, forming an *S*(6) ring motif (Table 1 ▸ and Fig. 1 ▸). 

## Supra­molecular features   

**Figure 2:**
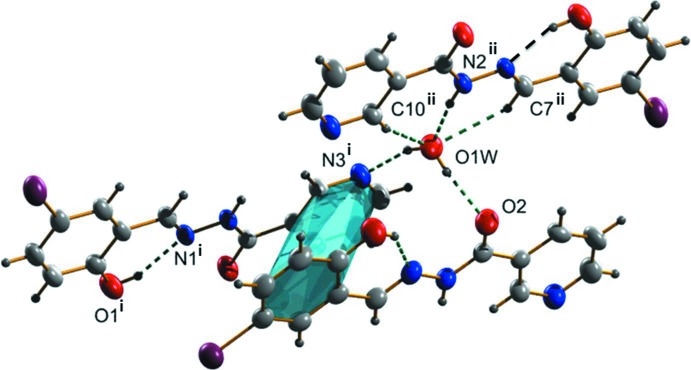
Hydrogen bonds (dashed lines) and a weak π–π inter­action (in blue) in the crystal of the title compound [symmetry codes: (i) *x*, −*y* + 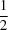, *z* + 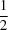; (ii) −*x*, *y* + 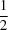, −*z* + 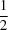].

**Figure 3:**
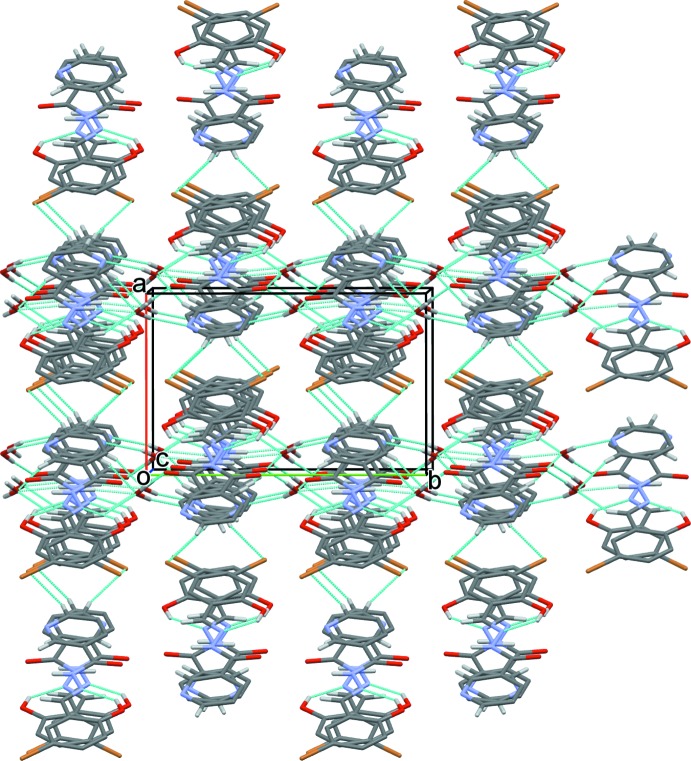
A view along the *c* axis of the crystal packing of the title compound. Hydrogen bonds are shown as dashed lines (see Table 1 ▸ for details) and H atoms not involved in hydrogen bonding have been omitted for clarity.

In the crystal, the water mol­ecule forms three hydrogen bonds with three different nicotinic hydrazone mol­ecules (Table 1 ▸ and Fig. 2 ▸). This compound is an example of a system where a single atom acts both as donor and acceptor. There are also C—H⋯O(water) contacts present enclosing 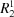
(6) and 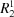
(7) ring motifs (Fig. 2 ▸). Finally sheets are formed lying parallel to (100). There are weak π–π inter­actions within the sheets involving the bromine-bearing aromatic ring of one mol­ecule and the pyridine ring of another, with a centroid–centroid distance of 3.8473 (15) Å (Fig. 2 ▸). The sheets are linked *via* C—H⋯Br inter­actions, forming a three-dimensional network (Table 1 ▸ and Fig. 3 ▸).

## Database survey   

A search of the Cambridge Structural Database (Version 5.36, update Feb. 2015; Groom & Allen, 2014 ▸) yielded 22 hits for the substructure *N*′-(2-hy­droxy­benzyl­idene)nico­tino­hydra­zide. The crystal structure of *N*′-(2-hy­droxy­benzyl­idene)nicotinohydrazide itself is reported as a monohydrate (IDASUB; Galić *et al.*, 2001 ▸), and the crystal structure of the chloro derivative of the title compound, which crystallized with two independent mol­ecules in the asymmetric unit, has also been reported (MOZPIB; Ren, 2009 ▸). In these two compounds, an intra­molecular O—H⋯N hydrogen bond is also present. The mol­ecules are also relatively planar, with the benzene and pyridine rings being inclined to one another by *ca* 4.2° in IDASUB, and by *ca* 12.8 and 1.9° in the two independent mol­ecules of MOZPIB. This last dihedral angle is similar to that in the title compound [*cf*. 0.74 (12)°]. In the crystal structure of *N*′-(2-hy­droxy­benzyl­idene)nico­tino­hydrazide monohydrate (IDASUB), the water mol­ecule forms three hydrogen bonds and is another example of a system where a single atom acts both as donor and acceptor.

## Synthesis and crystallization   

The title compound was prepared by adapting a reported procedure (Mathew & Kurup, 2011 ▸). A methano­lic solution of 5-bromo­salicyl­aldehyde (0.10051 g, 0.5 mmol) and nicotinic hydrazide (0.06857 g, 0.5 mmol) was refluxed for 3 h with two drops of glacial acetic acid. Light-yellow block-shaped crystals of the title compound were obtained by slow evaporation of the solvent. The crystals were filtered, washed with minimum qu­antity of methanol and dried over P_4_O_10_ *in vacuo* (yield: 0.22 g, 68.5%; m.p.: 480 K). Elemental analysis calculated for C_13_H_10_N_3_O_2_Br·H_2_O: C, 46.17, H, 3.58, N, 12.43%; found: C, 46.14, H, 3.57, N, 12.44%. IR FT–IR (KBr, cm^−1^) 3059 (NH), 3269(OH), 1680 (C=O), 1584 (C=N).

## Refinement   

Crystal data, data collection and structure refinement details are summarized in Table 2 ▸. The water, hydroxyl and NH H atoms were located in difference Fourier maps and refined with distances restraints: O—H = 0.86 (1) Å and N—H = 0.88 (1) Å. All C-bound H atoms were placed in calculated positions and refined as riding: C—H = 0.93 Å with *U*_iso_(H) = 1.2*U*_eq_(C). Three reflections were omitted owing to bad agreement, *viz*. 100, 110 and 200.

## Supplementary Material

Crystal structure: contains datablock(s) I. DOI: 10.1107/S2056989015009627/su5132sup1.cif

Structure factors: contains datablock(s) I. DOI: 10.1107/S2056989015009627/su5132Isup2.hkl

Supporting information file. DOI: 10.1107/S2056989015009627/su5132Isup3.cml

CCDC reference: 1401825

Additional supporting information:  crystallographic information; 3D view; checkCIF report

## Figures and Tables

**Table 1:** Hydrogen-bond geometry (, )

*D*H*A*	*D*H	H*A*	*D* *A*	*D*H*A*
O1H1N1	0.86(1)	1.91(2)	2.641(2)	143(3)
O1*W*H1*A*O2	0.85(1)	1.91(1)	2.756(2)	172(3)
O1*W*H1*B*N3^i^	0.85(1)	2.03(1)	2.845(3)	162(2)
N2H2O1*W* ^ii^	0.87(1)	1.95(1)	2.806(3)	169(3)
C7H7O1*W* ^ii^	0.93	2.49	3.263(3)	140
C10H10O1*W* ^ii^	0.93	2.45	3.362(3)	165
C11H11Br1^iii^	0.93	2.93	3.825(3)	162

Symmetry codes: (i) 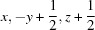; (ii) 
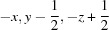
; (iii) 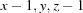.

**Table 2:** Experimental details

Crystal data	
Chemical formula	C_13_H_10_BrN_3_O_2_H_2_O
*M*_r_	338.17
Crystal system, space group	Monoclinic, *P*2_1_/*c*
Temperature (K)	296
*a*, *b*, *c* ()	8.1623(7), 12.5953(9), 13.2510(8)
()	90.226(3)
*V* (^3^)	1362.28(17)
*Z*	4
Radiation type	Mo *K*
(mm^1^)	3.03
Crystal size (mm)	0.42 0.12 0.11

Data collection	
Diffractometer	Bruker Kappa APEXII CCD
Absorption correction	Multi-scan (*SADABS*; Bruker, 2004 ▸)
*T*_min_, *T*_max_	0.349, 0.356
No. of measured, independent and observed [*I* > 2(*I*)] reflections	7570, 3331, 2233
*R*_int_	0.028
(sin /)_max_ (^1^)	0.668

Refinement	
*R*[*F* ^2^ > 2(*F* ^2^)], *wR*(*F* ^2^), *S*	0.036, 0.102, 0.99
No. of reflections	3331
No. of parameters	198
No. of restraints	5
H-atom treatment	H atoms treated by a mixture of independent and constrained refinement
_max_, _min_ (e ^3^)	0.46, 0.35

Computer programs: *APEX2*, *SAINT* and *XPREP* (Bruker, 2004 ▸), *SHELXS2014* (Sheldrick, 2008 ▸), *SHELXL2014* (Sheldrick, 2015 ▸), *ORTEP-3 for Windows* (Farrugia, 2012 ▸), *DIAMOND* (Brandenburg, 2010 ▸), *Mercury* (Macrae *et al.*, 2008 ▸), *PLATON* (Spek, 2009 ▸) and *publCIF* (Westrip, 2010 ▸).

## supplementary crystallographic information

### Crystal data

**Table:**

C_13_H_10_BrN_3_O_2_·H_2_O	*F*(000) = 680
*M**_r_* = 338.17	*D*_x_ = 1.649 Mg m^−^^3^
Monoclinic, *P*2_1_/*c*	Mo *K*α radiation, λ = 0.71073 Å
*a* = 8.1623 (7) Å	Cell parameters from 2267 reflections
*b* = 12.5953 (9) Å	θ = 2.9–25.5°
*c* = 13.2510 (8) Å	µ = 3.03 mm^−^^1^
β = 90.226 (3)°	*T* = 296 K
*V* = 1362.28 (17) Å^3^	Needle, yellow
*Z* = 4	0.42 × 0.12 × 0.11 mm

### Data collection

**Table:**

Bruker Kappa APEXII CCD diffractometer	2233 reflections with *I* > 2σ(*I*)
Radiation source: fine-focus sealed tube	*R*_int_ = 0.028
ω and φ scan	θ_max_ = 28.3°, θ_min_ = 3.0°
Absorption correction: multi-scan (*SADABS*; Bruker, 2004)	*h* = −10→4
*T*_min_ = 0.349, *T*_max_ = 0.356	*k* = −16→16
7570 measured reflections	*l* = −16→17
3331 independent reflections	

### Refinement

**Table:**

Refinement on *F*^2^	Hydrogen site location: mixed
Least-squares matrix: full	H atoms treated by a mixture of independent and constrained refinement
*R*[*F*^2^ > 2σ(*F*^2^)] = 0.036	*w* = 1/[σ^2^(*F*_o_^2^) + (0.0557*P*)^2^] where *P* = (*F*_o_^2^ + 2*F*_c_^2^)/3
*wR*(*F*^2^) = 0.102	(Δ/σ)_max_ < 0.001
*S* = 0.99	Δρ_max_ = 0.46 e Å^−^^3^
3331 reflections	Δρ_min_ = −0.35 e Å^−^^3^
198 parameters	Extinction correction: *SHELXL2014* (Sheldrick, 2015), Fc^*^=kFc[1+0.001xFc^2^λ^3^/sin(2θ)]^-1/4^
5 restraints	Extinction coefficient: 0.0129 (13)

### Special details

**Table:**

Geometry. All esds (except the esd in the dihedral angle between two l.s. planes) are estimated using the full covariance matrix. The cell esds are taken into account individually in the estimation of esds in distances, angles and torsion angles; correlations between esds in cell parameters are only used when they are defined by crystal symmetry. An approximate (isotropic) treatment of cell esds is used for estimating esds involving l.s. planes.

### Fractional atomic coordinates and isotropic or equivalent isotropic displacement parameters (Å^2^)

**Table:**

	*x*	*y*	*z*	*U*_iso_*/*U*_eq_	
C1	0.3276 (3)	0.35310 (19)	0.45911 (17)	0.0442 (6)	
C2	0.4115 (4)	0.3763 (2)	0.54807 (18)	0.0527 (7)	
H2	0.4272	0.4467	0.5667	0.063*	
C3	0.4712 (3)	0.2969 (2)	0.60856 (16)	0.0499 (6)	
H3	0.5276	0.3134	0.6676	0.060*	
C4	0.4471 (3)	0.19134 (19)	0.58114 (15)	0.0443 (6)	
C5	0.3653 (3)	0.16695 (19)	0.49313 (15)	0.0441 (6)	
H5	0.3499	0.0962	0.4753	0.053*	
C6	0.3057 (3)	0.24637 (18)	0.43065 (15)	0.0401 (5)	
C7	0.2191 (3)	0.21655 (19)	0.33854 (15)	0.0431 (6)	
H7	0.2084	0.1452	0.3219	0.052*	
C8	0.0128 (3)	0.32662 (17)	0.13365 (15)	0.0385 (5)	
C9	−0.0754 (3)	0.28892 (17)	0.04153 (15)	0.0358 (5)	
C10	−0.0990 (3)	0.18419 (18)	0.01526 (15)	0.0450 (6)	
H10	−0.0591	0.1321	0.0586	0.054*	
C11	−0.2332 (4)	0.2286 (2)	−0.12925 (19)	0.0560 (7)	
H11	−0.2880	0.2083	−0.1878	0.067*	
C12	−0.2160 (3)	0.3349 (2)	−0.10981 (18)	0.0580 (7)	
H12	−0.2581	0.3852	−0.1542	0.070*	
C13	−0.1355 (3)	0.36555 (19)	−0.02368 (18)	0.0487 (6)	
H13	−0.1214	0.4372	−0.0092	0.058*	
N1	0.1580 (2)	0.28694 (15)	0.28050 (12)	0.0426 (5)	
N2	0.0760 (3)	0.25244 (15)	0.19557 (13)	0.0403 (4)	
N3	−0.1758 (3)	0.15309 (16)	−0.06888 (13)	0.0524 (6)	
O1	0.2713 (3)	0.43501 (14)	0.40347 (14)	0.0634 (6)	
O2	0.0235 (3)	0.42215 (12)	0.15145 (12)	0.0586 (6)	
O1W	−0.0975 (3)	0.53043 (13)	0.31578 (13)	0.0641 (6)	
Br1	0.52655 (4)	0.08150 (2)	0.66553 (2)	0.06576 (15)	
H1	0.218 (4)	0.412 (2)	0.3516 (16)	0.079 (11)*	
H2'	0.075 (3)	0.1842 (9)	0.1847 (18)	0.060 (8)*	
H1A	−0.053 (4)	0.4947 (19)	0.2684 (15)	0.087 (11)*	
H1B	−0.129 (3)	0.4858 (16)	0.3591 (15)	0.065 (9)*	

### Atomic displacement parameters (Å^2^)

**Table:**

	*U*^11^	*U*^22^	*U*^33^	*U*^12^	*U*^13^	*U*^23^
C1	0.0439 (15)	0.0460 (13)	0.0426 (11)	−0.0079 (11)	0.0002 (10)	−0.0046 (10)
C2	0.0600 (18)	0.0505 (14)	0.0477 (13)	−0.0140 (13)	0.0000 (12)	−0.0154 (11)
C3	0.0470 (16)	0.0603 (15)	0.0423 (12)	−0.0117 (12)	−0.0076 (11)	−0.0122 (11)
C4	0.0385 (14)	0.0545 (14)	0.0399 (11)	0.0016 (11)	−0.0018 (10)	−0.0070 (10)
C5	0.0429 (15)	0.0446 (13)	0.0446 (12)	−0.0009 (11)	−0.0008 (10)	−0.0131 (10)
C6	0.0364 (13)	0.0462 (13)	0.0378 (11)	−0.0056 (10)	0.0010 (9)	−0.0113 (9)
C7	0.0467 (15)	0.0435 (13)	0.0391 (11)	−0.0050 (11)	−0.0024 (10)	−0.0104 (10)
C8	0.0475 (15)	0.0335 (12)	0.0346 (10)	−0.0053 (10)	0.0043 (9)	−0.0044 (8)
C9	0.0387 (13)	0.0339 (11)	0.0349 (10)	−0.0006 (9)	0.0038 (9)	−0.0037 (8)
C10	0.0599 (17)	0.0366 (12)	0.0384 (11)	−0.0042 (11)	−0.0085 (11)	−0.0012 (9)
C11	0.0588 (18)	0.0624 (17)	0.0469 (13)	−0.0008 (13)	−0.0150 (12)	−0.0026 (11)
C12	0.0626 (19)	0.0557 (17)	0.0556 (14)	0.0138 (14)	−0.0201 (13)	0.0040 (11)
C13	0.0533 (17)	0.0363 (13)	0.0564 (14)	0.0067 (11)	−0.0034 (12)	0.0000 (10)
N1	0.0479 (13)	0.0449 (11)	0.0348 (9)	−0.0106 (9)	−0.0022 (8)	−0.0090 (8)
N2	0.0510 (13)	0.0350 (11)	0.0348 (9)	−0.0064 (9)	−0.0039 (8)	−0.0069 (7)
N3	0.0667 (16)	0.0464 (12)	0.0439 (11)	−0.0042 (11)	−0.0110 (10)	−0.0066 (9)
O1	0.0871 (16)	0.0443 (11)	0.0587 (11)	−0.0099 (9)	−0.0178 (11)	−0.0052 (8)
O2	0.0956 (17)	0.0328 (9)	0.0472 (9)	−0.0063 (8)	−0.0065 (10)	−0.0094 (6)
O1W	0.1112 (19)	0.0316 (9)	0.0494 (10)	0.0065 (10)	0.0025 (11)	0.0023 (8)
Br1	0.0773 (3)	0.0634 (2)	0.0564 (2)	0.00894 (15)	−0.01868 (14)	−0.00546 (12)

### Geometric parameters (Å, º)

**Table:**

C1—O1	1.348 (3)	C8—C9	1.492 (3)
C1—C2	1.392 (3)	C9—C10	1.378 (3)
C1—C6	1.407 (3)	C9—C13	1.384 (3)
C2—C3	1.370 (4)	C10—N3	1.336 (3)
C2—H2	0.9300	C10—H10	0.9300
C3—C4	1.392 (3)	C11—N3	1.327 (3)
C3—H3	0.9300	C11—C12	1.370 (4)
C4—C5	1.376 (3)	C11—H11	0.9300
C4—Br1	1.892 (2)	C12—C13	1.370 (3)
C5—C6	1.386 (3)	C12—H12	0.9300
C5—H5	0.9300	C13—H13	0.9300
C6—C7	1.457 (3)	N1—N2	1.378 (2)
C7—N1	1.274 (3)	N2—H2'	0.872 (10)
C7—H7	0.9300	O1—H1	0.858 (10)
C8—O2	1.229 (2)	O1W—H1A	0.854 (10)
C8—N2	1.345 (3)	O1W—H1B	0.845 (9)
			
O1—C1—C2	117.9 (2)	N2—C8—C9	117.42 (18)
O1—C1—C6	122.8 (2)	C10—C9—C13	117.5 (2)
C2—C1—C6	119.3 (2)	C10—C9—C8	125.3 (2)
C3—C2—C1	121.0 (2)	C13—C9—C8	117.22 (19)
C3—C2—H2	119.5	N3—C10—C9	123.8 (2)
C1—C2—H2	119.5	N3—C10—H10	118.1
C2—C3—C4	119.6 (2)	C9—C10—H10	118.1
C2—C3—H3	120.2	N3—C11—C12	123.4 (2)
C4—C3—H3	120.2	N3—C11—H11	118.3
C5—C4—C3	120.1 (2)	C12—C11—H11	118.3
C5—C4—Br1	120.11 (18)	C13—C12—C11	118.7 (2)
C3—C4—Br1	119.75 (17)	C13—C12—H12	120.6
C4—C5—C6	120.9 (2)	C11—C12—H12	120.6
C4—C5—H5	119.6	C12—C13—C9	119.4 (2)
C6—C5—H5	119.6	C12—C13—H13	120.3
C5—C6—C1	119.1 (2)	C9—C13—H13	120.3
C5—C6—C7	118.8 (2)	C7—N1—N2	117.49 (18)
C1—C6—C7	122.1 (2)	C8—N2—N1	117.59 (18)
N1—C7—C6	120.9 (2)	C8—N2—H2'	125.4 (19)
N1—C7—H7	119.5	N1—N2—H2'	116.9 (19)
C6—C7—H7	119.5	C11—N3—C10	117.2 (2)
O2—C8—N2	122.4 (2)	C1—O1—H1	111 (2)
O2—C8—C9	120.1 (2)	H1A—O1W—H1B	106 (2)
			
O1—C1—C2—C3	179.6 (2)	N2—C8—C9—C10	0.7 (3)
C6—C1—C2—C3	−0.7 (4)	O2—C8—C9—C13	3.2 (3)
C1—C2—C3—C4	−0.4 (4)	N2—C8—C9—C13	−177.9 (2)
C2—C3—C4—C5	0.8 (4)	C13—C9—C10—N3	0.1 (4)
C2—C3—C4—Br1	−179.05 (19)	C8—C9—C10—N3	−178.6 (2)
C3—C4—C5—C6	−0.1 (4)	N3—C11—C12—C13	0.0 (5)
Br1—C4—C5—C6	179.72 (18)	C11—C12—C13—C9	−0.6 (4)
C4—C5—C6—C1	−1.0 (3)	C10—C9—C13—C12	0.6 (4)
C4—C5—C6—C7	−179.7 (2)	C8—C9—C13—C12	179.4 (2)
O1—C1—C6—C5	−179.0 (2)	C6—C7—N1—N2	−179.09 (19)
C2—C1—C6—C5	1.4 (4)	O2—C8—N2—N1	−1.4 (3)
O1—C1—C6—C7	−0.3 (4)	C9—C8—N2—N1	179.77 (18)
C2—C1—C6—C7	−180.0 (2)	C7—N1—N2—C8	−179.3 (2)
C5—C6—C7—N1	177.9 (2)	C12—C11—N3—C10	0.7 (4)
C1—C6—C7—N1	−0.8 (3)	C9—C10—N3—C11	−0.7 (4)
O2—C8—C9—C10	−178.1 (2)		

### Hydrogen-bond geometry (Å, º)

**Table:**

*D*—H···*A*	*D*—H	H···*A*	*D*···*A*	*D*—H···*A*
O1—H1···N1	0.86 (1)	1.91 (2)	2.641 (2)	143 (3)
O1*W*—H1*A*···O2	0.85 (1)	1.91 (1)	2.756 (2)	172 (3)
O1*W*—H1*B*···N3^i^	0.85 (1)	2.03 (1)	2.845 (3)	162 (2)
N2—H2′···O1*W*^ii^	0.87 (1)	1.95 (1)	2.806 (3)	169 (3)
C7—H7···O1*W*^ii^	0.93	2.49	3.263 (3)	140
C10—H10···O1*W*^ii^	0.93	2.45	3.362 (3)	165
C11—H11···Br1^iii^	0.93	2.93	3.825 (3)	162

Symmetry codes: (i) *x*, −*y*+1/2, *z*+1/2; (ii) −*x*, *y*−1/2, −*z*+1/2; (iii) *x*−1, *y*, *z*−1.
